# Effect of fruits granola (Frugra®) consumption on blood pressure reduction and intestinal microbiome in patients undergoing hemodialysis

**DOI:** 10.1038/s41440-024-01895-1

**Published:** 2024-09-19

**Authors:** Hajime Nagasawa, Shogo Suzuki, Takashi Kobayashi, Tomoyuki Otsuka, Teruyuki Okuma, Satoshi Matsushita, Atsushi Amano, Yoshio Shimizu, Yusuke Suzuki, Seiji Ueda

**Affiliations:** 1 Department of Nephrology, Juntendo University Faculty of Medicine, Tokyo, Japan; 2 Division of Nephrology, Department of Internal Medicine, Juntendo University Shizuoka Hospital, Shizuoka, Japan; 3 Department of Granola Health Care and Preventive Medicine, Juntendo University Faculty of Medicine, Tokyo, Japan; 4 Division of Kidney Health and Aging, the Center for Integrated Kidney Research and Advance, Shimane University Faculty of Medicine, Shimane, Japan; 5 Department of Radiological Technology, Juntendo University Faculty of Health Science, Tokyo, Japan; 6 Department of Cardiovascular Surgery, Juntendo University Faculty of Medicine, Tokyo, Japan

**Keywords:** Blood pressure, Fruits granola, Indoxyl sulfate, Microbiome

## Abstract

Cardiovascular diseases (CVDs) are a major cause of death in patients undergoing hemodialysis (HD). Blood pressure (BP) and uremic toxins are well-known risk factors for CVDs, which are influenced by diet. Dietary fiber supplementation in patients undergoing HD may reduce the risk of CVDs by improving lipid profiles and inflammatory status and lowering the levels of the uremic toxin indoxyl sulfate (IS). In this study, we investigated the relationship between the intestinal microbiota and risk factors for CVDs, such as BP and serum IS, in patients undergoing HD who consumed fruits granola (FGR). The study participants were selected from patients undergoing HD at the Izu Nagaoka Daiichi Clinic and consumed FGR for 2 months. Body composition and blood samples were tested at months 0, 1, 2 and fecal samples were collected at months 0 and 2 for intestinal microbiota analysis. FGR consumption decreased systolic and diastolic BP, estimated salt intake, and serum IS levels and improved the stool characteristics according to the Bristol Stool Form Scale (*N* = 24). Gut microbiota analysis showed an increase in the alpha diversity and abundance of *Blautia* and *Neglecta*. The abundance of lactic acid- and ethanol-producing bacteria also significantly increased, whereas the abundance of indole-producing bacteria significantly decreased. FGR consumption could be a useful tool for salt reduction, fiber supplementation, and improvement of the intestinal environment, thus contributing to improvement of BP and the reduction of other risk factors for CVDs in patients undergoing HD.

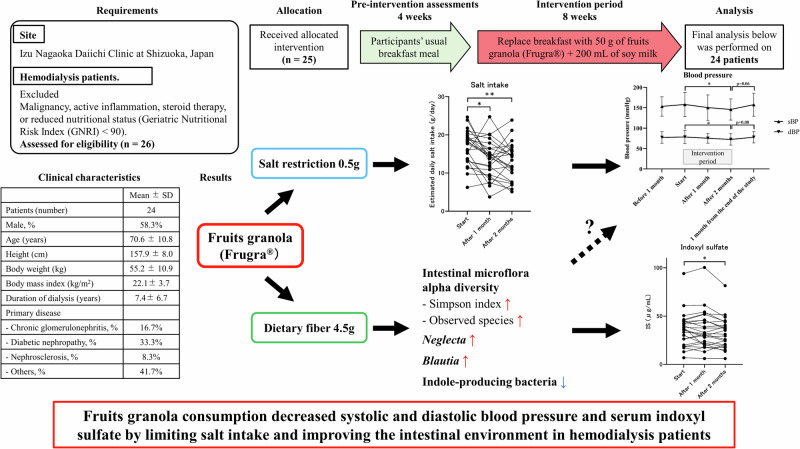

## Introduction

The number of patients undergoing hemodialysis (HD) in Japan has exceeded 300,000 and continues to increase [1]. Patients with chronic kidney disease (CKD) were reported to have progressive atherosclerotic lesions from the preservation phase before the initiation of dialysis, and the mortality rate by cardiovascular diseases (CVDs) was reported to be higher as renal dysfunction progresses [2]. The risk of CVD-related death is approximately 10 times higher in patients undergoing HD than in healthy controls [3]. Many risk factors for CVDs are attributable to diet and lifestyle, including hypertension, obesity, diabetes, lipid metabolism, and smoking [4]. Furthermore, in patients with renal diseases such as CKD and patients undergoing dialysis, blood levels of indoxyl sulfate (IS) and p-cresyl sulfate, uremic toxins derived from intestinal bacteria, are high and correlate with the incidence of CVDs and mortality [5, 6]. These uremic toxins originate from indole, p-cresol, and trimethylamine, which are produced by intestinal microbiota metabolism when dietary protein components such as tryptophan, tyrosine, carnitine, and choline are not fully absorbed in the small intestine and reach the colon [7]. Therefore, diet is one of the most important factors in the prognosis of patients undergoing dialysis.

Patients undergoing dialysis are subjected to strict dietary restrictions ranging from daily caloric intake and electrolyte mass to fluid intake [8]. Specifically, patients undergoing HD are at risk of hypertension, which is thought to be caused at least in part by sodium intake and fluid retention. Salt restriction in patients undergoing HD may improve blood pressure (BP) control and weight gain during HD, leading to more favorable outcomes [9, 10]. Therefore, the dietary standards for patients undergoing HD set a recommended daily salt intake of <6 g [8].

Constipation was also reported to occur more frequently in patients undergoing HD, with 63.1% experiencing it [11]. Patients undergoing HD have to limit their intake of fiber-rich foods (daily fiber intake of 5.9 g/day) such as vegetables, fruits, and seaweeds to prevent hyperkalemia [12]. This may explain the high prevalence of constipation in these patients. Indeed, supplementation with fermented fiber has been suggested to improve the lipid profile, decrease systemic inflammatory conditions, and reduce the risk of CVDs in patients undergoing HD [13]. Another report suggested that increasing dietary fiber in these patients may reduce plasma concentrations of the colon-derived solutes IS and p-cresol sulfate without enhancing therapy [14]. We have previously confirmed the safety of a product rich in dietary fibers, i.e., fruits granola (FGR, Frugra®, Calbee Inc., Ltd. Tokyo, Japan), in a pilot study of 11 patients undergoing HD. Moreover, we previously reported a decrease in BP, improvement in the Bristol Stool Form Scale (BSS), and a decrease in serum IS levels [15]. A study analyzing defecation and intestinal microbiota showed that FGR consumption increased the defecation frequency in healthy Japanese individuals and the abundance levels of *Parabacteroides*, a short-chain fatty acid (SCFA)-producing bacterium, and *Ruminiclostridium 5*, which is associated with the recovery of circadian rhythm disturbances [16]. In our pilot study, we hypothesized that the supplementation of dietary fibers such as beta-glucan, which is the main ingredient of FGR and present in oats, may also contribute to the improvement of intestinal microbiota; however, we have not analyzed this in detail [15]. Therefore, in the present study, we examined the relationship between gut microbiota and risk factors for CVDs, such as BP and serum IS, in patients undergoing HD who consumed FGR for 8 weeks instead of their regular breakfast.

## Materials and methods

### Participants

Among patients undergoing HD at the Izu Nagaoka Daiichi Clinic, 26 patients indicated their willingness to participate in this study. However, patients undergoing HD who presented with malignancies, had active inflammation, were on steroid therapy, or had reduced nutritional status (Geriatric Nutritional Risk Index [GNRI] <90) were excluded.

All participants provided written informed consent for study participation. The Ethics Committee of Juntendo University approved this study. This study was managed according to the principles of the Declaration of Helsinki (approval number: 17-247). Moreover, the study protocol was registered with the University Hospital Medical Information Network Clinical Trial Registry (Registration number: UMIN000031666).

### Clinical and biochemical parameter measurements

All clinical and biochemical parameters were measured as described in previous studies. BP was measured noninvasively at the brachial artery on the non–fistula arm, and predialysis BP was measured after a 5-minites rest. Before the start of the HD session, blood samples were obtained from the arterial HD line. Then, the serum was immediately frozen at −80 °C until analysis. The serum levels of IS, which is a representative gut-derived uremic toxin, were determined by internal-surface reversed-phase high-performance liquid chromatography (Fushimi Pharmaceutical Co., Ltd., Kagawa, Japan) [17]. Interleukin-6 (IL-6), tumor necrosis factor-α (TNF-α), and high-sensitivity C-reactive protein (Hs-CRP) were measured by standard laboratory methods such as at SRL Inc. (Tokyo, Japan). Other blood biochemistry values were measured using standard laboratory methods [18]. The nutritional status was assessed using the GNRI, which was calculated using the following formula: GNRI = [1.489 × albumin (g/dl)] + [41.7 × (body weight/ideal body weight)]. The ideal body weight of patients was calculated according to their height. Furthermore, body composition parameters such as bone mineral content, total body volume, body fat percentage, and total body water were measured by bioelectrical impedance analysis (InBody 470, InBody, Cerritos, CA, USA). For bowel health, the participants answered a questionnaire about bowel movements. We also checked any changes in the frequency of stool or the form of stool according to the BSS [19]. Moreover, IS levels were measured before and after the intervention to assess the intestinal status.

### Study procedures

**Fig. 1:**
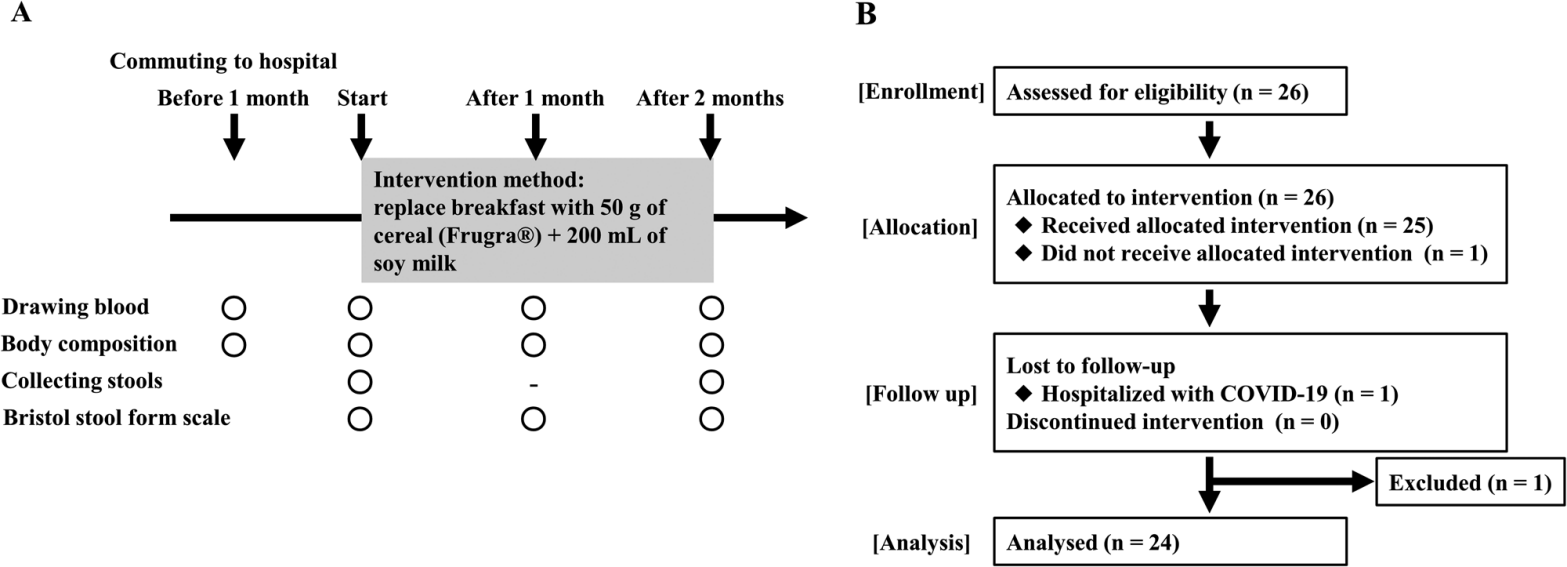
Study schedule and flow diagram of the study. **A** In this study, participants were recruited from patients who were undergoing HD at the Izu Nagaoka Daiichi Clinic, Shizuoka, Japan, and who consumed FGR for 2 months after a 1-month preobservation period. **B** Flow diagram of this study. A total of 26 patients undergoing HD participated, and 24 of them were included in the analysis. FGR fruits granola, HD hemodialysis

Participants’ daily breakfast was substituted with FGR for 8 weeks, which was 4 weeks after preintervention evaluations. Laboratory tests and body composition assessments were performed after every 4 weeks (Fig. 1A). FGR is commonly served with 200 mL of milk or yogurt. In this study, the participants were served FGR mixed with 200 mL of soymilk to prevent phosphorus overload (Supplementary Table 1).

### Estimated daily salt intake using Watson’s formula

Estimates of daily salt intake were calculated using the Watson’s formula [20].

Salt intake (g/day) = [salt intake during dialysis (g)]/[days on dialysis] + 0.04/BW.

Salt intake during dialysis (g) = Na intake (mmol)*58.5/1 000.

Na intake (mmol) = (Vw + ⊿BW)*Cs − Vw*Ce.

Vw (men) = (2.447 − 0.09516 Y + 0.1074 H + 0.3362 BW)* 1 000/BW.

Vw (women) = ( − 2.097 + 0.1069 H + 0.2466 BW)*1 000/BW.

Cs, Na before dialysis on Wednesday; Ce, Na after dialysis on Monday; Y, years; H, height; BW, body weight after dialysis on Monday.

### Microbiome analysis

Stool sample collection, DNA extraction, and 16S rRNA (variable regions, V1–V3) gene sequence analysis using the MiSeq system (Illumina, San Diego, CA, USA) were performed according to the methods described by Hatayama et al. [21]. Alpha diversity analyses were performed as mentioned previously [21]. The intestinal microbiota was compared using ALDEx2 as described by Kono et al. [22]. Centered log ratio-transformed intestinal microbiota data were used for the ALDEx2 comparison.

### Statistics

Continuous data are presented as mean ± standard deviation (SD), whereas categorical data are presented as frequencies and percentages. Within-group comparisons of gut microbiota were conducted using the paired *t* test or Wilcoxon signed-rank test, depending on the data distribution; p-values were corrected for multiple testing using the Benjamini–Hochberg method. Paired *t* test was performed using the t test function with paired = TRUE. Benjamini–Hochberg multiple test correction was performed using the p.adjust function with method = BH. The threshold for statistical significance was set at *p* < 0.05. Clinical characteristics, BP, brain natriuretic peptide (BNP) levels, estimated daily salt intake, and serum IS were statistically analyzed using GraphPad Prism version 8.4.3. (GraphPad Software Inc., San Diego, CA, USA). The D’Agostino–Pearson test was used to examine the normal distribution of the data, and the Bartlett test was used to examine the variance of the data. The one-way repeated analysis of variance was used for BP analysis, and Tukey’s test was used for multiple comparisons. The Friedman test, a nonparametric test, was then used, and Dunn’s multiple-comparison test was performed as a posteriori analysis. Data are presented as mean ± SD, and the significance level was set at *p* < 0.05.

## Results

### Study flow diagram and clinical characteristics of patients undergoing HD

A patient who had severe anemia symptoms was excluded from the study (Fig. 1B). Another patient who had COVID-19 during the study was also withdrawn from the analysis. Therefore, the final analysis included 24 patients. The clinical characteristics of the included patients undergoing HD are shown in Table 1.

**Table 1:** Clinical characteristics of patients undergoing hemodialysis

	Mean ± SD
Patients (number)	24
Male, % (number)	58.3% (14)
Age (years)	70.6 ± 10.8
Height (cm)	157.9 ± 8.0
Body weight (kg)	55.2 ± 10.9
Body mass index (kg/m^2^)	22.1 ± 3.7
Duration of dialysis (years)	7.4 ± 6.7
Primary disease	
-Chronic glomerulonephritis, % (number)	16.7% (4)
-Diabetic nephropathy, % (number)	33.3% (8)
-Nephrosclerosis, % (number)	8.3% (2)
-Others, % (number)	41.7% (10)

Data are shown as mean ± standard deviation (SD)

### Metabolic parameters and body composition of patients undergoing HD

All 24 participants who completed the study did not show any adverse effects, and no changes in electrolyte levels due to FGR consumption were observed during the study period (Supplementary Table 1). Because FGR contains high amounts of iron, it was expected to improve anemia levels; however, no changes in hemoglobin levels or ferrokinetics were noted during the observation period. Because oats also contain beta-glucan, they were expected to affect the lipid profile of the patients; however, no changes in low-density lipoprotein, high-density liproprotein, or triglycerides were found. Inflammatory markers such as Hs-CRP and IL-6 were also not affected by FGR consumption.

Body composition measured using InBody indicated that none of the parameters changed during the study period (Supplementary Table 2). These results suggest the safety of FGR consumption in patients undergoing HD.

### BP, BNP, and estimated daily salt intake of patients undergoing HD

**Fig. 2:**
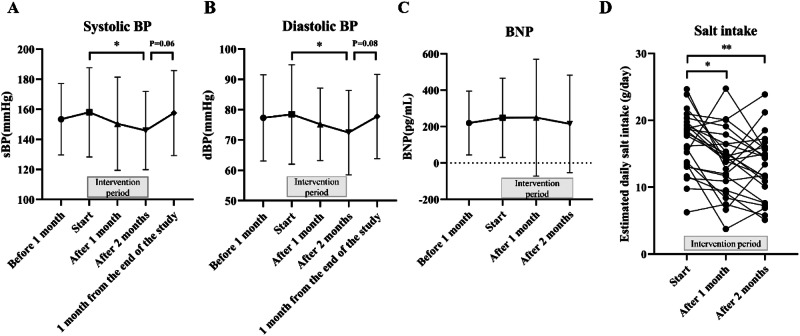
BP, BNP, and estimated daily salt intake in patients undergoing HD. **A** Systolic BP, (**B**) Diastolic BP changes from 1 month prior to the start of the study to 1 month from the end of the study, and (**C**) BNP changes from 1 month prior to the start of the study to the end of the FGR consumption period. **D** Changes in estimated daily salt intake during the study period. Mean ± standard deviation (SD), one-way repeated-measures ANOVA test with Tukey test and Friedman test with Dunn’s multiple comparison post hoc test, * *p* < 0.05, ** *p* < 0.01. BNP brain natriuretic peptide, BP blood pressure, HD hemodialysis, FGR fruits granola

FGR consumption significantly reduced systolic BP (sBP) levels from 158.0 ± 29.7 mmHg at the start to 145.9 ± 26.0 mmHg after 2 months (Fig. 2A, Supplementary Table 3). Similarly, diastolic BP (dBP) significantly decreased from 78.4 ± 16.4 mmHg at the start to 72.5 ± 14.0 mmHg after 2 months of FGR consumption (Fig. 2B, Supplementary Table 3). In addition, the sBP and dBP were 157.5 ± 28.3 and 77.8 ± 13.9 mmHg, respectively, at 1 month from the end of the FGR intervention study, both tended to increase compared with measurements taken at 2 months from the start of the study. However, FGR consumption did not cause significant changes in BNP levels (Fig. 2C, Supplementary Table 3). The estimated salt intake significantly decreased from 16.7 ± 4.5 g/day at the start to 13.0 ± 4.9 g/day after 2 months (Fig. 2D, Supplementary Table 4). The results indicate that FGR consumption may reduce salt intake in patients undergoing HD and thereby help in lowering BP. Some patients have decreased estimated salt intake while others have not (Fig. 2D). There were not any differences in the various parameters between these patients (Data not shown).

### Bowel health of patients undergoing HD

**Fig. 3:**
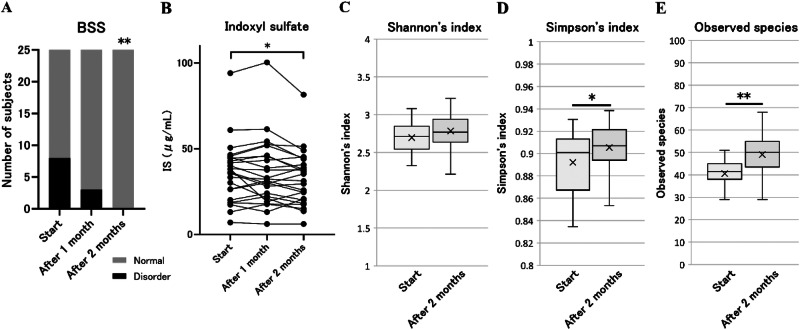
BSS, IS, and intestinal microflora alpha diversity in patients undergoing HD. **A** Changes in BSS during the FGR consumption period: normal, BSS 3–5; abnormal, BSS 1, 2, 6, and 7. A chi-square test was performed for the number of participants with normal stools (vs start), **p < 0.01. **B** Changes in the serum IS during the FGR consumption period. Serum IS concentration (μg/mL), mean ± SD. Friedman test with Dunn’s multiple comparison post hoc test (vs start), *p < 0.05. Changes in intestinal microbiota alpha diversity at the start and the end of FGR consumption: (**C**) Shannon’s index, (**D**) Simpson’s index, and (**E**) Observed species. BSS Bristol Stool Form Scale, FGR fruits granola, HD hemodialysis, IS indoxyl sulfate

FGR consumption improved stool characteristics according to the BSS (Fig. 3A, Supplementary Table 5). Serum IS levels significantly decreased from 36.2 ± 18.0 µg/mL at the start to 32.9 ± 16.0 µg/mL after 2 months (Fig. 3B, Supplementary Table 6).

In the gut microbiota analysis, the Shannon index showed no difference after FGR consumption; however, the Simpson index and the alpha diversity of the observed species significantly increased (Fig. 3C–E). FGR consumption significantly increased the abundance of lactic acid- and ethanol-producing bacteria but significantly decreased the abundance of indole-producing bacteria (Table 2). No significant changes were found in the abundance of the other 11 bacterial genera. The absolute effect size of the seven genera was >0.2 (Table 3), and of the seven genera, the abundance levels of *Blautia* and *Neglecta* were significantly increased by FGR consumption. These findings suggest that FGR consumption may improve the intestinal environment.

**Table 2:** Metabolite-producing bacteria in patients undergoing hemodialysis

Metabolite-producing bacteria	Relative abundance (mean value, %)		Difference value
	Start	After 2 months	[Start → After 2 months]
Butyric acid-producing *bacteria*	15.65	17.06	1.42
Acetic acid-producing *bacteria*	41.13	43.82	2.68
Propionic acid-producing *bacteria*	4.96	5.40	0.44
Lactic acid-producing *bacteria*	37.15	40.58	3.43*
Equol-producing *bacteria*	0.58	0.41	−0.17
Vitamin B1-producing *bacteria*	6.69	6.66	−0.03
GABA-producing *bacteria*	17.48	17.28	−0.19
Serotonin-producing *bacteria*	6.31	7.55	1.24
Dopamine-producing *bacteria*	6.07	7.52	1.46
Ammonia-producing bacteria	0.09	0.01	−0.08
Indole-producing bacteria	0.90	0.60	−0.30*
Hydrogen sulfide-producing *bacteria*	1.96	1.74	−0.22
Histamine-producing *bacteria*	6.31	7.56	1.25
Ethanol-producing *bacteria*	12.92	15.35	2.43**

Statistical significance compared with start, *p < 0.05, **p < 0.01

*GABA* gamma amino butyric acid

**Table 3:** Abundance of intestinal bacteria (genus level) in patients undergoing hemodialysis

Genus of *bacteria*	Relative abundance (mean value, %)		Retention rate (%)		Start vs After 2 months
	Start	After 2 months	Start	After 2 months	Effect size
*Neglecta*	0.23	0.77	45.8	66.7	0.44*
*Blautia*	4.82	6.09	100.0	100.0	0.44*
*Agathobaculum*	0.10	0.23	33.3	58.3	0.26
*Intestinibacter*	0.29	0.35	33.3	58.3	0.23
*Roseburia*	0.65	1.07	66.7	83.3	0.22
*Coprobacillus*	0.22	0.04	33.3	25.0	−0.22
*Mediterraneibacter*	0.57	0.88	58.3	79.2	0.20

Statistical significance compared with start, *p < 0.05

### Administration of antihypertensive medications during the study period

In this study, medications were managed to maintain optimal BP control. As a result, the dose of antihypertensive medication was increased in one participant, whereas they were reduced or discontinued in four participants during the study period (Supplementary Table 7), further suggesting the blood pressure lowering effect of FGR.

## Discussion

This study noted the following salient findings of FGR consumption in patients undergoing HD. (1) FGR comsumption significantly decreased systolic and diastolic BP, estimated salt intake, and serum IS levels. (2) FGR consumption significantly improved stool characteristics according to the BSS. (3) Intestinal microbiota analysis showed a significant increase in alpha diversity (Simpson index and observed species). (4) The abundance of lactate- and ethanol-producing bacteria significantly increased, whereas that of indole-producing bacteria significantly decreased. (5) Genus-level analysis showed a significant increase in the abundance levels of *Blautia* and *Neglecta*.

In patients undergoing HD, high salt intake leads to fluid retention, which increases the incidence of CVDs such as hypertension, left ventricular hypertrophy, and cardiovascular death [23]. Limiting salt intake is a major therapeutic goal for patients undergoing HD, set at <6 g/day, but is currently not achieved [24]. In this study, participants’ salt intake before the start of the observation was 16.7 ± 4.5 g/day, which is a significant deviation from the guideline target. A study also reported that salt intake in Shizuoka Prefecture was higher than the national average for men and women, which is thought to be due to regional differences in diet [25]. In the present study, the estimated salt intake in patients with anuria was calculated using Watson’s formula. In an earlier report, the estimated daily salt intake was calculated from urinary sodium excretion, and differences in calculation methods may have contributed to the discrepancy [26]. FGR consumption for breakfast reduced the salt intake to 13.0 ± 4.9 g/day at 2 months from FGR intervention. A previous study suggested that the 3.7 g/day reduction in salt intake by FGR consumption may reduce BP levels (Fig. 2A, B, D). Low salt intake may be useful in reducing the risk of CKD progression [27], and salt intake reduction by FGR consumption may be a useful tool for patients undergoing HD. However, because a 1 g/day reduction in salt intake was reported to be associated with a 1 mmHg reduction in sBP [28], the systolic and diastolic BP were reduced by −12.1 and −5.9 mmHg, respectively, compared with the 3.7 g/day reduction in salt intake achieved in this study, suggesting that factors other than salt may be involved. Seasonal variability in BP has been observed, and in Japan, BP was reported to be the highest in March but the lowest in August [29]. This study was conducted from January to March; although the seasonal variation data showed an increase in sBP from 125.8 to 127.0 mmHg, the decrease in sBP and dBP in March, 2 months after the FGR intervention, indicated that FGR had a certain effect. In addition, April, which corresponds to 1 month from the end of the study, was 126.4 mmHg, indicating a decrease in values in the seasonal variation data, both sBP and dBP in our study tended to increase in April, reflecting that FGR may have had some effects on BP (Fig. 2A, B, Supplementary Table 3). However, this study was conducted only from January to March, which is one of the study limitations. Therefore, it is desirable to consider it at other times in the future.

Next, we examined the relationship of BP with intestinal microflora; *Blautia* is a SCFA-producing bacterium, which has been reported to decrease in patients with hypertension [30]. A study also reported that Japanese patients with diabetes have less *Blautia* than healthy individuals and that the administration of *Blautia wexlerae* to mice improves insulin resistance [31]. Therefore, in this study, *Blautia* was significantly increased in patients undergoing HD and may have contributed to the decrease in BP via the improvement of insulin resistance. CKD-induced hypertension was correlated with a modified gut microbiota profile and dysregulated renal SCFA receptor expression. Interestingly, the abundance of *Blautia*, a genus of beneficial bacteria, was increased by resveratrol and resveratrol butyrate ester treatment in adenine-treated CKD rats [32]. Arterial hypertension was reported to be associated with high *Blautia* levels. Moreover, a combination of a high-fat diet and high *Blautia* levels was a very common factor in patients with type 2 diabetes mellitus [33]. Whether *Blautia* contributes to high or low BP awaits future reports. *Neglecta* is a genus reclassified in 2016 from *Clostridium sporosphaeroides*, and few studies have reported the association of *Neglecta* with products and human health status [34]. Therefore, the effect of a significant increase in *Neglecta* on patients undergoing dialysis is currently unknown. The presence of butyrate-producing *Roseburia* was reported to be decreased in patients with hypertension [35]. Similarly, a study comparing hypertension and normotension reported an enriched abundance of *Roseburia hominis* in the normotensive group [36]. In the present study, FGR consumption increased the abundance of *Roseburia*, suggesting that the increased presence of *Roseburia* may contribute to the improvement of hypertension. High salt intake causes dysbiosis of the intestinal microbiota, resulting in decreased production of SCFA and abnormal BP regulation via membrane receptors such as GPR43, GPR41, and Olfr78, resulting in hypertension [37]. In addition, decreased SCFA production induces an inflammatory response, also resulting in hypertension [38]. A previous study reported that feeding mice a high-salt diet altered the composition of the intestinal microbiota and significantly reduced the abundance of *Lactobacillus murinus*, a type of lactobacillus; however, the administration of *Lactobacillus murinus* prevents the worsening of salt-induced hypertension [39]. Other studies have reported that oxidative stress resulting from high salt intake contributes to hypertension development [37, 40, 41]. Endothelial cell dysfunction is a characteristic feature of salt-sensitive hypertension and is associated with the inability to upregulate nitric oxide (NO) production in response to high salt intake [37, 42]. High salt levels result in enhanced sodium entry into endothelial cells via ENaC and the activation of NADPH oxidase [37, 43]. In other words, the FGR-induced decrease in BP might be due to the reduced salt intake and improvement of intestinal microflora. Furthermore, various bacterial species have been reported to be associated with hypertension. The results of meta-analysis showed that Shannon index, one of the microbiome alpha diversity factors, significantly decreased and Firmicutes/Bacteroidetes (F/B) ratio significantly increased in patients with hypertension compared with healthy controls [44]. The increase was accompanied by an increase in the abundance levels of *Prevotella_9*, *Megasphaera*, *Parasutterella*, and *Escherichia_Shigella*, whereas those of *Bacteroides* and *Faecalibacterium* decreased [45]. A meta-analysis targeting the Chinese population indicated that patients with hypertension suffer from impaired gut microbial diversity, manifesting as an increased F/B ratio, increased abundance levels of phylum *Firmicutes* and genera *Megasphaera*, *Escherichia, Shigella*, and *Klebsiella*, and decreased abundance levels of *Bacteroidaceae*, *Bifidobacterium*, *Faecalibacterium*, *Roseburia*, and *Ruminococcus* [46–48]. In an Australian population, the abundance of *Bacteroidetes*, *Prevotella spp*., *Alistipes spp*., *Firmicutes*, *Clostridium spp*., and *Lactobacillus spp* increased, and gut microbial richness and evenness were downregulated in patients with hypertension [46, 49]. Other studies have found a significant increase in the abundance of *Prevotella* in patients with hypertension [46, 50]. The abundance of *Clostridium perfringens 1*, *Romboutsia*, *Ruminococcus 2*, and *Intestinibacter* negatively correlated with sBP and dBP in patients with hypertension receiving antihypertensive treatment [46, 51]. A study reported that salt sensitivity was related to intestinal bacteria in addition to genetic background [52]. FGR certainly reduced estimated salt intake, however, in the supplementary table 2, FGR did not alter body weight or body fluid volume. Improvement of gut bacteria and vascular tone may have influenced the improvement of hypertension. Although the relationship between hypertension and intestinal microbiota has been reported in various ways, the sample size was not enough to discuss them in this study; thus, further large-scale studies are needed to confirm the present findings.

Alpha diversity is one of the most important factors when considering the state of the intestinal microbiota. Alpha diversity is a numerical expression of the abundance of species in a sample; the number of OTUs, Chao1, is an estimate of the total number of species in a sample, and the Shannon index considers the evenness of the bacterial species into account [53]. A previous study revealed that alpha diversity, bacterial abundance, and diversity indices were decreased in patients with diabetic kidney disease (DKD) [54]. In CKD, impaired renal function causes the accumulation of high levels of uremic toxins, which then reach the intestine and instigate modifications in bacterial composition and fecal metabolite profile [55]. Dietary fiber intake is important to increase the diversity of the intestinal microbiota. Dietary fibers can be insoluble and soluble, and the gut microbiota feed on soluble fiber [56]. Oats, the main ingredient in FGR, contain high levels of the soluble fiber beta-glucan, which reaches the large intestine, feeds the intestinal microflora, and increases the amount of metabolite SCFAs [57]. Furthermore, the produced SCFAs are expected to affect other intestinal microflora and contribute to the increasing diversity of intestinal microflora [55]. In other words, the supplementation of soluble dietary fibers from the beta-glucan of oats in FGR may have contributed to the improvement of the intestinal environment by increasing alpha diversity. In addition, previous studies have reported that increasing fiber intake in patients undergoing HD may reduce the plasma concentrations of IS and p-cresol sulfate, uremic toxins derived from enteric bacteria, without enhancing dialysis therapy [14]. Similar to our previous findings [15], FGR consumption also reduced serum IS levels in the present study. These results may indicate that fiber intake decreased the abundance of indole-producing *bacteria* in the intestinal tract and suppressed indole production, leading to a decrease in serum IS levels (Table 2, Fig. 3B). Although previous studies have shown the usefulness of a gum arabic fiber in patients with CKD and resistant starch in patients undergoing HD [14, 58, 59], FGR contains multiple whole grains, including oats, rye, and brown rice, which are expected to have additive and synergistic effects given their respective soluble dietary fibers [16]. In addition, given the differences in intestinal microflora among individuals and different intestinal bacteria have different dietary fibers that are compatible with feeding [60], FGR, which contains various dietary fibers, may be beneficial in improving the intestinal microflora [61]. Polyphenols in grains and fruits have also been reported to be beneficial to the intestinal microbiota. This may be a unique advantage of FGR. The relationship between IS and protein intake was also reported, that is, an increase in IS depending on the dietary protein intake was confirmed in patients undergoing HD [62]. On the contrary, in the previous study, dietary intake of tryptophan may not be a determinant of the IS levels in patients with CKD and HD [63]. Another study reported that a vegetarian diet reduced IS concentrations. Although this could be due to differences in dietary protein intake, a vegetarian diet may reduce IS production by the intestinal microbiome [64]. Dietary intake in patients undergoing HD was suggested to be related to IS and gut microbiome health; thus, dietary content may be an important indicator.

Constipation is a frequent complication in patients undergoing HD because dietary fiber intake is limited to prevent hyperkalemia. Constipation must be resolved in patients undergoing HD because it leads to deterioration of the intestinal environment and accumulation of uremic toxins. In this study, in addition to normal stools in all 24 participants, the abundance of lactic acid-producing *bacteria* also increased, suggesting that lactic acid may have a bowel-regulating effect by increasing peristalsis of the intestinal tract (Table 2) [65, 66]. This factor may explain why supplementation with dietary fiber, which is lacking in patients undergoing dialysis, results in an improved intestinal environment. IS and p-cresyl sulfate concentrations were reported to be negatively correlated with fiber intake in patients undergoing HD [67]. Previous studies have suggested that supplementation with fermentable fiber may improve the lipid profile, decrease systemic inflammation, and reduce the risk of CVDs in patients undergoing HD [13]. Dietary fiber supplementation may be one of the most important nutrients because this intervention helps in improving stool properties, lowering uremic toxins in the intestinal tract, and suppressing CVDs development. Prebiotics (partially hydrolyzed guar gum) and synbiotics (*Bifidobacterium longum* and partially hydrolyzed guar gum) improved microbiota and SCFAs in Japanese patients undergoing HD [68, 69]. Thus, further detailed research on FGR consumption would confirm its effects for salt reduction, fiber supplementation, and improvement of intestinal health, thus contributing to the reduction of risk factors for CVDs in patients undergoing HD.

This study has several limitations. First, because this study was conducted in Japanese individuals and the intervention period was only 8 weeks, its application to a racial group other than the Japanese population and its contribution to a long-term treatment strategy are unclear. Second, this study was conducted only from January to March. Therefore, it is desirable to conduct related studies at other times in the future. The population has a very high salt intake, which indicates the regional characteristics of Shizuoka; thus, testing the results in other populations may be necessary. Third, because previous studies were referenced in the study design, data such as salt intake, blood sample, and intestinal bacteria at 1 month before the start of the study and 1 month from the end of the study were unavailable. Fourth, the placebo group could not be set because multiple components in FGR were involved. Future research may be considerd incorporating these indicators into the study design to properly determine the effect of the test food. Soymilk consumption may have a favorable effect on BP levels [70]. Fifth, because we do not know the dietary contents of the participants during the study period, we have not considered the relationship between protein intake and IS, and whether intake of calorie, electrolytes, minerals, etc., are related to lower BP and IS and changes in the intestinal microbiota. In other words, future trials keeping a record of meals may make deeper observations. Sixth, patients undergoing HD often have complications associated with other diseases and are likely to be taking various medications. Although several medications may change the intestinal environment, we have not examined each medication separately. Seventh, the relationship between gut microbiota and prognosis in patients undergoing HD is still poorly understood; thus, further studies are needed to determine the changes associated with long-term FGR consumption and the incidence of CVDs.

## Conclusions

FGR consumption could be a useful means for salt reduction, fiber supplementation, and intestinal environment improvement by decreasing the abundance levels of indole-producing bacteria and increasing those of *Blautia* and *Neglecta*, thus contributing to the reduction of risk factors for CVDs in patients undergoing HD such as hypertension and increase in serum IS levels by dysbiosis.

## Supplementary information

Supplementary Information
