# Supplementary material for: Effect of fruits granola (Frugra®) consumption on blood pressure reduction and intestinal microbiome in patients undergoing hemodialysis
Source: Hypertens Res. 2024 Sep 19;47(11):3214–24. doi: 10.1038/s41440-024-01895-1 (PMC11534689; doi:10.1038/s41440-024-01895-1)
Supplement: Supplementary file 1 — Supplementary Information [file 41440_2024_1895_MOESM1_ESM.docx]

**Supplementary Table 1. Laboratory data of patients undergoing hemodialysis.**

|  | Before 1 month | Start | After 1 month | After 2 months | 1 month from the end of the study |
| --- | --- | --- | --- | --- | --- |
| Sodium (mEq/L) | 139.2 ± 3.1 | 138.8 ± 2.8 | 139.5 ± 2.3 | 138.2 ± 2.6 | 139.0 ± 3.0 |
| Potassium (mEq/L) | 4.9 ± 0.7 | 5.0 ± 0.8 | 5.0 ± 0.6 | 4.9 ± 0.7 | 4.8 ± 0.7 |
| Calcium (mg/dL) | 9.1. ± 0.6 | 8.9 ± 0.8 | 8.9 ± 0.7 | 9.0 ± 0.7 | 8.9 ± 0.8 |
| Phosphorus (mEq/L) | 4.9 ± 1.5 | 4.7 ± 1.3 | 4.7 ± 1.1 | 4.7 ± 0.8 | 4.9 ± 1.1 |
| Hemoglobin (g/dL) | 11.2 ± 1.0 | 11.0 ± 0.8 | 10.8 ± 0.7 | 11.0 ± 0.7 | 10.8 ± 0.7 |
| Iron (μg/dL) | 63.0 ± 31.7 | 61.5 ± 25.6 | 59.9 ± 20.4 | 57.2 ± 18.0 | 57.8 ± 17.8 |
| Ferritin (ng/mL) | 52.5 ± 34.1 | 49.7 ± 30.9 | 53.8 ± 39.6 | 47.3 ± 33.3 | 54.9 ± 43.2 |
| Albumin (g/dL) | 3.7 ± 0.3^*^ | 3.5 ± 0.3 | 3.6 ± 0.3^*^ | 3.7 ± 0.3^**^ | 3.6 ± 0.3^*^ |
| Blood glucose (mg/dL) | 113.3 ± 27.3 | 111.6 ± 25.7 | 116.6 ± 39.7 | 116.2 ± 31.5 | 110.5 ± 24.4 |
| LDL cholesterol (mg/dL) | 81.3 ± 23.7 | 79.0 ± 22.9 | 81.6 ± 23.8 | 80.7 ± 24.9 | 78.8 ± 22.2 |
| HDL cholesterol (mg/dL) | 52.7 ± 16.0 | 52.8 ± 16.9 | 55.0 ± 16.4 | 55.2 ± 16.8 | 53.8 ± 16.1 |
| Triglyceride (mg/dL) | 95.3 ± 40.2 | 110.5 ± 64.9 | 113.1 ± 52.7 | 120.2 ± 71.7 | 116.0 ± 64.5 |
| High-sensitivity CRP (mg/dL) | 0.23 ± 0.40 | 0.14 ± 0.17 | 0.14 ± 0.16 | 0.15 ± 0.17 | No data |
| IL-6 (pg/mL) | 6.7 ± 5.8 | 6.4 ± 3.3 | 7.0 ± 7.3 | 6.2 ± 5.1 | No data |

Data are shown as mean ± standard deviation. Differences were analyzed by the one-way analysis of variance. Statistical significance compared with start, *p < 0.05, **p < 0.01. CRP, C-reactive protein; HDL, high density lipoprotein; IL-6, interleukin 6; LDL, low-density lipoprotein; TNF-α, tumor necrosis factor-α.

**Supplementary Table 2. Body composition of patients undergoing hemodialysis**

|  | Start | After 1 month | After 2 months | 1 month from the end of the study |
| --- | --- | --- | --- | --- |
| Total body water (L) | 31.0 ± 5.4 | 30.9 ± 6.1 | 31.1 ± 5.6 | No data |
| Protein (kg) | 8.1 ± 1.4 | 8.1 ± 1.5 | 8.0 ± 1.5 | No data |
| Minerals (kg) | 2.8 ± 0.5 | 2.8 ± 0.5 | 2.8 ± 0.5 | No data |
| Body fat mass (kg) | 14.0 ± 6.3 | 13.9 ± 6.7 | 14.4 ± 6.9 | No data |
| Soft lean mass (kg) | 39.5 ± 6.9 | 39.5 ± 7.2 | 39.0 ± 7.1 | No data |
| Body mass index (kg) | 22.5 ± 3.7 | 22.4 ± 3.8 | 22.4 ± 3.8 | No data |
| Body fat (%) | 24.4 ± 8.6 | 24.2 ± 9.2 | 25.0 ± 9.5 | No data |
| Dry weight (kg) | 55.2 ± 10.9 | 55.2 ± 10.7 | 55.1 ± 10.5 | 55.1 ± 10.5 |
| Increasing body weight per day (kg) | 2.1 ± 0.7 | 2.0 ± 0.6 | 2.1 ± 0.7 | 2.2 ± 0.6 |
| Increasing body weight per 2 days (kg) | 2.9 ± 0.8 | 2.8 ± 1.1 | 2.7 ± 0.7 | 3.0 ± 0.8 |

Data are shown as mean ± standard deviation. Differences were analyzed by the one-way analysis of variance.

**Supplementary Table 3. Blood pressure and brain natriuretic peptide levels of patients undergoing hemodialysis**

|  | Before 1 month | Start | After 1 month | After 2 months | 1 month from the end of the study |
| --- | --- | --- | --- | --- | --- |
| Systolic blood pressure (mmHg) | 153.4 ± 23.7 | 158.0 ± 29.7 | 150.4 ± 30.9 | 145.9 ± 26.0 * | 157.5 ± 28.3 |
| Diastolic blood pressure (mmHg) | 77.3 ± 14.2 | 78.4 ± 16.4 | 75.2 ± 11.9 | 72.5 ± 14.0 * | 77.8 ± 13.9 |
| Brain natriuretic peptide (pg/mL) | 219.6 ± 175.2 | 248.4 ± 217.8 | 249.5 ± 320.7 | 215.1 ± 268.1 | No data |

Data are shown as mean ± standard deviation. Differences were analyzed by the one-way analysis of variance. Statistical significance compared with start, *p < 0.05.

**Supplementary Table 4. Estimated daily salt intake of patients undergoing hemodialysis.**

|  | Start | After 1 month | After 2 months |
| --- | --- | --- | --- |
| Estimated daily salt intake (g/day) | 16.7 ± 4.5 | 13.7 ± 4.9^*^ | 13.0 ± 4.9^**^ |

Data are shown as mean ± standard deviation. Daily salt intake was estimated by Watson’s formula. Differences were analyzed by the one-way analysis of variance. Statistical significance compared with start, *p < 0.05, **p < 0.01.

**Supplementary Table 5. Bristol Stool Form Scale in patients undergoing hemodialysis**

|  | Start | After 1 month | After 2 months |
| --- | --- | --- | --- |
| Number of participants with abnormal forms | 7 | 3 | 0 |
| Number of participants with normal forms | 17 | 21 | 24* |

Values present the number of participants. A chi-square test was performed for participants with abnormal BSS forms and those with normal forms. Statistical significance compared with start, *p < 0.05. Abnormal forms: BSS 1, 2, 6, and 7. Normal forms: BSS 3–5. BSS, Bristol Stool Form Scale.

**Supplementary Table 6. Indoxyl sulfate in patients undergoing hemodialysis**

|  | Start | After 1 month | After 2 months |
| --- | --- | --- | --- |
| Indoxyl sulfate (μg/mL) | 36.2 ± 18.0 | 35.7 ± 19.8 | 32.9 ± 16.0* |

Data are shown as mean ± standard deviation. Differences were analyzed by the one-way analysis of variance. Statistical significance compared with start, *p < 0.05.

**Supplementary Table 7. Record of changes in antihypertensive medications taken by patients undergoing hemodialysis**

| Subject No. | Change in antihypertensive medications |
| --- | --- |
| 1 |  |
| 2 |  |
| 3 |  |
| 4 |  |
| 5 |  |
| 6 | Discontinue telmisartan 20 mg/day at 8 weeks after FGR intervention. |
| 7 |  |
| 8 | Dose up carvedilol to 20 mg/day from 10 mg/day at 6 weeks after FGR intervention. |
| 9 | Dose down nifedipine CR to 40 mg/day from 80 mg/day at 2 weeks after FGR intervention. |
| 10 |  |
| 11 |  |
| 12 |  |
| 14 | Discontinue bisoprolol 5 mg/day at 4 weeks after FGR intervention. |
| 15 |  |
| 16 |  |
| 17 |  |
| 18 |  |
| 19 |  |
| 20 |  |
| 21 |  |
| 22 |  |
| 23 | Discontinue carvedilol 2.5 mg/day at 2 weeks from the end of the study. |
| 24 |  |
| 25 | Dose down methyldopa to 500 mg/day from 750 mg/day at 5 weeks after FGR intervention and dose down methyldopa to 375 mg/day from 500 mg /day at 7 weeks after FGR intervention. |
